# Artificial Intelligence and Blockchain: How Should Emerging Technologies Be Governed?

**DOI:** 10.3389/frma.2022.801549

**Published:** 2022-02-11

**Authors:** Cornelius Kalenzi

**Affiliations:** Korea Policy Center for the Fourth Industrial Revolution (KPC4IR), Korea Advanced Institute of Science and Technology, Daejeon, South Korea

**Keywords:** artificial intelligence, blockchain, governance, innovation policy, AI ethics, AI principles

## Abstract

Governing emerging technologies is one of the most important issues of the twenty-first century, and primarily concerns the public, private, and social initiatives that can shape the adoption and responsible development of digital technologies. This study surveys the emerging landscape of blockchain and artificial intelligence (AI) governance and maps the ecosystem of emerging platforms within industry and public and civil society. We identify the major players in the public, private, and civil society organizations and their underlying motivations, and examine the divergence and convergence of these motivation and the way they are likely to shape the future governance of these emerging technologies. There is a broad consensus that these technologies represent the present and future of economic growth, but they also pose significant risks to society. Indeed, there is also considerable confusion and disagreement among the major players about navigating the delicate balance between promoting these innovations and mitigating the risks they pose. While some in the industry are calling for self-regulation, others are calling for strong laws and state regulation to monitor these technologies. These disagreements, are likely to remain for the foreseeable future and may derail the optimal development of governance ecosystems across jurisdictions. Therefore, we propose that players should consider erecting new safeguards and using existing frameworks to protect consumers and society from the harms and dangers of these technologies. For instance, through re-examining existing legal and institutional arrangements to check whether these cater for emerging issues with new technologies, and as needed make necessary update/amendments. Further, there may be cases where existing legal and regulated systems are completely outdated and can't cover for new technologies, for example, when AI is used to influence political outcomes, or crypto currency frauds, or AI-powered autonomous vehicles, such cases call of agile governance regimes. This is important because different players in government, industry, and civil are still coming to terms with the governance challenges that these emerging technologies pose to society, and no one has a clear answer on optimal way to promote these technologies, at the same time limit the dangers they pose to users.

## Introduction

Fourth Industrial Revolution (4IR) technologies, such as blockchain and artificial intelligence (AI), are being adopted rapidly by industry and governments worldwide, and consumers use services that depend on these technologies every day. The increased adoption of blockchain and AI has led to far-reaching changes in every aspect of human life—from getting a job to keeping it; from how people connect and with whom to how people date; from how the police keep us safe to who gets imprisoned and who gets released, among many other use cases (McKinsey Company, 2019)—disrupting the business processes of traditional industries in almost every sector (Quintais et al., 2019).

However, the increased adoption of these digital technologies has also introduced new and unprecedented challenges, such as privacy breaches, digital fraud, new forms of money laundering, intensified bias and discrimination, new safety and liability issues, technology-related unemployment, expanded surveillance, the potential for destructive robot-powered (Dempsey, 2020) wars, and market polarization (Acemoglu and Restrepo, 2020), among others. These technologies have opened a Pandora's Box of legal, ethical, policy, regulatory, and governance issues currently being grappled with by many actors in the government, industry, and civil society.

Thus, the question of governing^1^ disruptive digital technologies (we broadly define governance as methods of monitoring technologies by the public and private sectors to promote potential digital innovations and mitigate the risks posed by these innovations), has become one of the most important questions of the twenty-first century (Winfield and Jirotka, 2018). Governance is not just about mitigating the risks of emerging technologies. It is also about making changes to ensure that emerging technologies can be legally deployed and their full potentials maximized. Since 2017, we have witnessed a rising global debate (Pagallo, 2018) in private, public, academic, and social arenas on the governance of digital technologies (Winfield and Jirotka, 2018). During this period, for example, civil society and inter-governmental platforms have published over 200 AI principles that ask AI developers to adopt “ethics by design principles,” while regulators are expressing strong concerns about potential abuse of blockchain-powered applications, such as crypto assets.

Within industry, there is an ongoing debate between entrepreneurs and shareholders and “BigTech firms, with some calling for regulation of digital technologies (BBC, 2020), while, the other tech firms, are keen to protect their innovations from stifling regulations. Many tech firms have published their own ethical principles (Jobin et al., 2019) and established ethics committees. Additionally, the Partnership on AI (PAI) and the Blockchain Association—inter-industry associations advocating for responsible development of emerging technologies—were launched recently as attempts to promote self-regulation and influence public policy so that innovations can thrive.

Moreover, the public sector is responding to a varied mixture of governance frameworks aimed at promoting digital innovations and guarding against risks they may pose to society (OECD, 2019). In the past 2 years alone, almost all major countries have published digital policies and investment plans aimed at promoting digital innovations. Furthermore, some, such as South Korea and the UK, have experimented with regulatory sandboxes (regulatory tools that permit innovators to experiment and introduce their innovations to the market under minimum and controlled regulations and supervision) (Financial Conduct Authority, 2015). During the same period, countries have issued over 450 regulatory proposals targeting digital innovations, more than 200 in Europe alone (Hogan Lovells, 2019). A contradiction or paradox is apparent in governments wanting to invest in digital innovations while at the same time expecting to regulate digital technologies, instilling a growing sense of confusion about how best to govern these emerging technologies across all major jurisdictions.

Amid this debate, only few studies have focused on emerging platforms for digital innovation governance. Some scholars have focused more on the ethical governance of emerging technologies. For example, Winfield and Jirotka (2018) proposed a framework that guides the ethical governance of AI and robotics, showing how ethics feed into standards, which, in turn, lead to regulations (Winfield and Jirotka, 2018). Furthermore, Cath (2018) studied the question of governing high-risk technologies and the appropriate frameworks, and provided suggestions on digital technology governance. Another expert, Etzioni^2^, proposed a framework of three rules for AI regulation: AI should be subjected to existing laws, which must be updated to suit AI systems; AI systems should fully disclose that they are not human; and AI systems must not keep or publish users' private information without their explicit permission. Similarly, Takanashi et al. (2020) called for multiple stakeholders to establish governance mechanisms for emerging technologies, and (Feijóo et al., 2020) highlighted emerging platforms and called for technology diplomacy. Their contributions are important in answering critical questions about emerging technology governance.

However, we still find that the role of emerging technology governance platforms in the public, private, and civil society arenas, and how they are already interacting (working for, with, and against each other) in governing digital technologies is poorly understood. There is a broad agreement that the public and private sectors as well as civil society need to work together. The missing puzzle is how this can be realized in practice. As above, some argue for self-regulation, how this can be realized is not yet clear. For example, should the public sectors trust the private sector that relies emerging technologies such as AI and Blockchain to self-regulate? Others argue for a strong public hand in governing of emerging technologies, they suggest that most private sector players especially big tech will not self-regulate without a strong government hand (Kwoka and Valletti, 2021). However, they may be overestimating the ability or “power” of governments (most) to have say in the emerging technology governance debate. The reality is that majority of governments may not have the ability, knowhow, resources to effectively govern, regulate emerging technologies (Cusumano et al., 2021). Then how about the civil society players? Do they have impactful say on shaping the rules to govern emerging technologies?

This study argues that the forces shaping digital technology governance across different jurisdictions emerge from the governance struggles and evolution of these emerging platforms. Therefore, we map out the ecosystem of emerging platforms in the public, civil society, and private sectors to improve our overall understanding of emerging technology governance. We seek to identify the major players and their underlying motivations in the new digital technologies' governance space, outline where these motivations differ and where they converge (see outline in Table 1 above). Finally, we consider (preliminarily) their relative strength and power to shape how emerging technologies are governed. Through this approach, this study contributes to the governance debate by attempting to provide realistic view each major player and their role in shaping governance of emerging technologies. In the following sections, we examine some of the emerging platforms and perspectives of the players.

**Table 1:** Emerging technology governance ecosystem map.

**Level**	**Stakeholder**	**Role**	**Motivation**
Market and industry	Big-Tech firms	They make AI and blockchain applications and new digital services	Ambition and profit
	Entrepreneurs and start-ups	They innovate AI and blockchain applications	Stakeholders dividends, profit
	Consumers	They use digital products and services and provide data	Efficiency and cost
	Investors	They provide seed capital to fund digital innovations	Financial returns
Civil society	Non-government standards bodies	They set standards and principles for responsible adoption of digital technologies	Mission
	Non-government organizations	They are advocates, watchdogs and quasi-regulators	Mission and human rights
	Activist academics	Watchdogs and provide knowledge base on ethical issues around emerging technologies	Driven by intellectual curiosity
	Non–profits; media platforms	They put a spotlight on the digital industry by highlighting the good, bad, and ugly	Mission
Public sector	Regulatory bodies	They investigate and punish regulatory breaches	Public safety, efficiency, cost, consumer protection
	Innovation investors	They invest in digital innovation through R&D, capacity building, entrepreneurship funds, etc.	Country mission
	Governance councils	They coordinate efforts for responsible adoption of AI	Innovation and ethics
	Multinational bodies	They set policies and standards	Innovation
	Advisory councils	They advise governments and policies and strategies	Mission

### Defining Emerging Technology Governance Platforms

Throughout this article, we have introduced the concept of emerging technology governance “platforms” and by this, we specifically refer to an organization or grouping of organizations from either the public, private or civil society that may use “their organization base/platform or group voice/platform to push for policies, or standards or laws, or principles, or voice opinions, etc., regarding the governance of emerging technologies. This definition should not be confused with AI-powered digital platforms (e.g., Facebook, Google) or Blockchain-powered platforms, e.g., Binance that focuses on helping to facilitate interactions across a large number of participants, using modern software, hardware, and networking technologies (Cusumano et al., 2019).

The later are now dominated by global tech behemoths whose technologies and platforms are used by billions of users across the globe, facilitating speed and convenience in interactions, whether it is financial transactions across borders, buy and selling of products and services or personal connections. Much as such platforms have enormous benefits to users, they also pose risks such as privacy breaches, fraud, and cyber security breaches, etc., which can instantly affect millions of users and impact can reverberate to far flung of the world. For example, a privacy breach at Facebook or Google can instantly affect users across the Globe. Similarly, crypto frauds and scams such as the recent “Squid Game crypto” (BBC, 2021b) and hundreds of others such scams affect small investors from South Korea to Uganda.

## Emerging Approaching To Governing The Fourth Industrial Revolution

**Figure 1:**
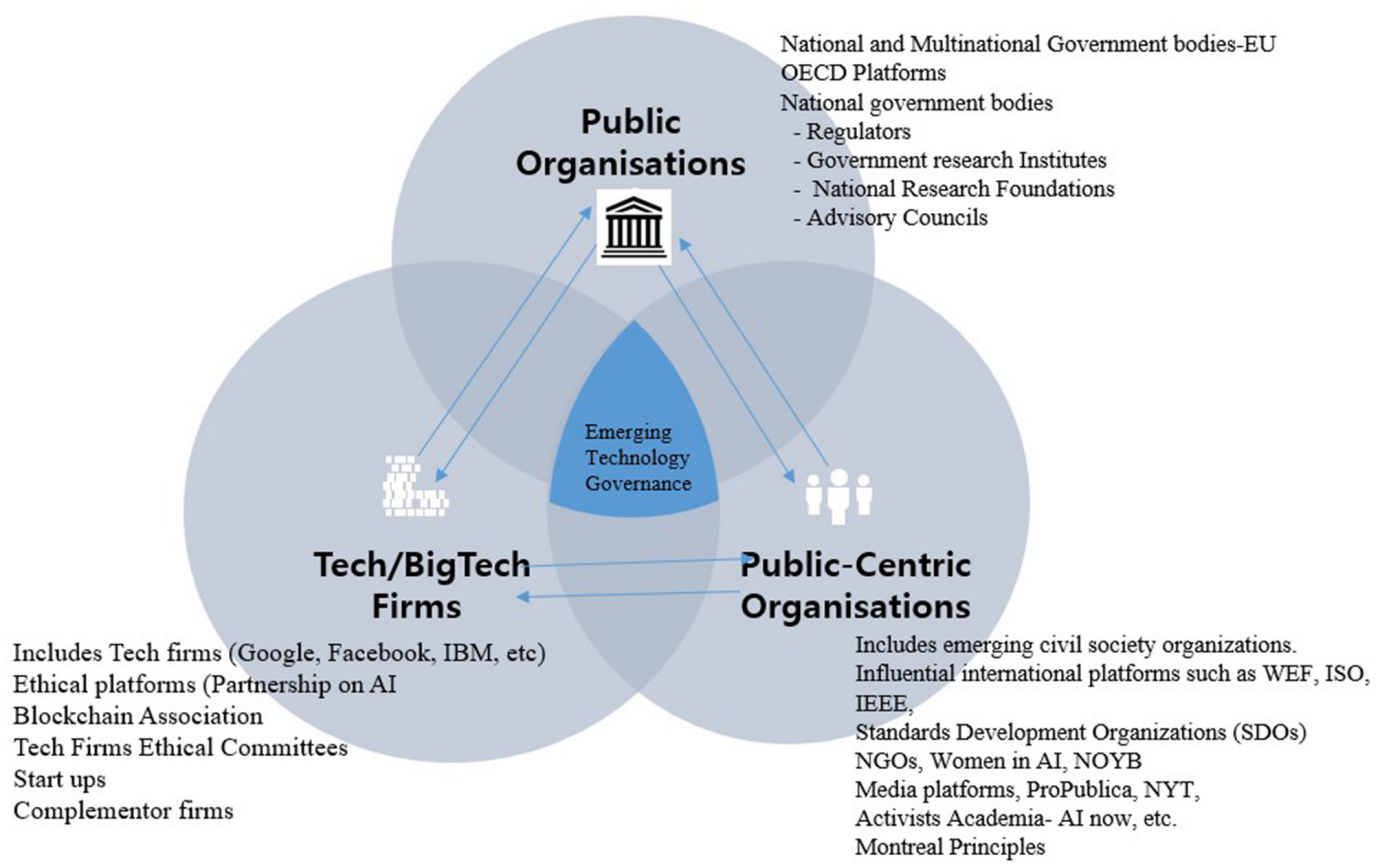
Types of emerging technology governance.

In Figure 1 below, we propose a simple analytical model adapted from Lynn (2010) to provide a descriptive understanding of the many layers and players in the governance of emerging technologies.

### Emerging Technology Governance Centered in the Market

**Figure 2:**
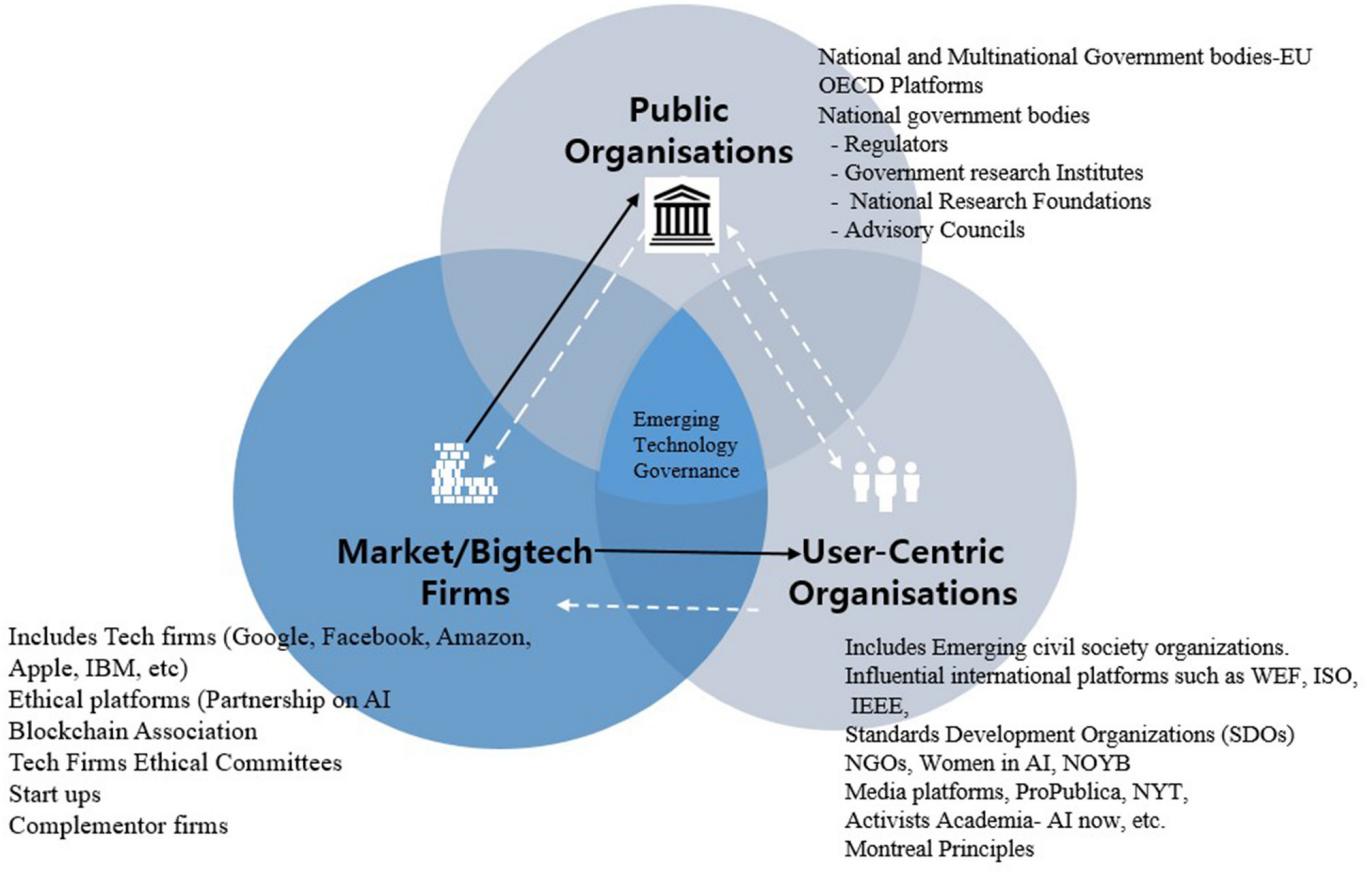
Technology governance centered in the markets.

In this governance model (Figure 2), the institutional platforms for the governance of emerging technologies are dictated by the market or industry, while public sector players (regulators) and civil society have a limited role in issues of governance and innovation promotion. This model prevails in countries such as the US, where the IT industry, in general, has a long history of self-/light regulation. In this model, industry, especially US BigTech, is primarily motivated by profit, and the profit lens plays a dominant role in shaping technology governance. Firms view emerging technologies as “real-world applications technology, which is part of every fabric of society,^3^” and are capable of transforming industries using smarter algorithms and big data. These firms also recognize that new technologies are a source of business and competitive advantage that, however, may pose significant risks to users.

Tech firms have adopted various approaches to harness the business potential of emerging technologies and deal with the numerous challenges they face. The first and most common approach focuses on first deploying and marketing emerging technologies, and deals with ethical, legal, and governance issues later. In other words, this approach prioritizes business first and ethics second (Murgia and Shrikanth, 2019). We look at a recent case of AI-powered smart speakers to illustrate how this practice works. A UN report and a number of consumer complaints have been leveled against these “sexist and discriminatory speakers” (Rawlinson, 2019), which have nevertheless been released to the market; indeed, aside from some typically minimizing public relations responses, firms manufacturing, and selling these products have barely addressed the ethical issues raised by them. There are similar cases in the blockchain context, where products and services such as initial coin offerings (ICOs) of cryptocurrencies have turned out to offer only fraudulent, valueless tokens. This model of putting profits before ethical considerations has forced regulators to take reactive measures. Experts argue, however, that it is difficult for regulators to come up with comprehensive and adequate measures, beyond issuing warnings and guidance, because of the global nature of digital technologies (Takanashi et al., 2020). Similar issues have included fights between Google and its employees over the company's plans to make contentious technologies, and Amazon's plans to sell facial recognition software to governments even after stakeholders and employees have raised ethical issues (Waters, 2018). The participation of these companies in profitable but ethically questionable projects reinforces the narrative that industry is not serious about ethical and social issues (Gregg and Greene, 2019). In this view, their recent governance initiatives represent only “ethics washing”; their focus will always be on profits first and ethics later.

However, ethical issues continue to raise concerns among corporate customers as well (e.g., a survey by Deloitte in 2018 of 1,400 executives showed that 32% were concerned about ethical issues around emerging technologies). Thus, major tech firms, including IBM, Microsoft, Amazon, and Google, have started embracing “ethical and responsible” platforms for technology governance. For example, in 2016, they established a powerful inter-industry association called the PAI and the Blockchain Association for responsible innovations. However, industrial experts, such as Yochai (2019) and Bengio (see Benkler 2019; Castelvecchi, 2019), have interpreted such moves as attempts to create lobbying platforms to influence the formation of rules that govern emerging technologies (Simonite, 2019) and as attempts to avoid regulation (Wagner, 2018). While the industry argues that self-regulation is preferable because regulation stifles innovation, the strategy of “ethics washing” (Peukert and Kloker, 2020) and lobbying are leading to fears of regulatory inertia and failure to address the fundamental challenges and issues posed by unaccountable digital technologies.

In addition, tech firms have established technology ethics committees and principles as platforms for shaping the governance of emerging technologies and addressing issues of fairness, safety, privacy, transparency, inclusiveness, and accountability. However, many experts suggest that industry efforts fall short of showing that these firms are committed to addressing the emerging ethical and governance issues. Critics say that, at best, the industry is engaging in ethics washing (Wagner, 2018, 6–7), pointing to the fact that the principles proposed by tech firms are not binding and there are no mechanisms or frameworks to ensure that they are implemented. The ethical committees are just advisory, and have no power to ensure that their advice is adhered to by the companies. Moreover, there is no verification/auditing mechanisms of ethics adherence. Thus far, the examples of Amazon and Google moving ahead in contentious projects despite protests from employees and stakeholders confirm critics' fears that the tech firms lack any genuine commitment to addressing ethical and governance issues.

Further, tech firms are shaping governance rules through International Telecommunication Union (ITU) standards platforms (Gross et al., 2019). For example, recent reports indicate that Chinese AI firms, such as ZTE and Zahua, are working with the ITU to propose standards for AI-powered facial recognition technologies. The ability to influence standards gives companies not only a competitive and market advantage over others but also the ability to influence the technology policies adopted by countries, especially in the developing world. Thus, the fact that AI firms are writing standards for technologies that have proven to be contentious globally (especially in the US, China, and the UK) should be considered with caution. Such efforts offer tech firms a chance to self-regulate and set rules with minimal oversight from civil society^4^ and consumer advocacy agencies—two constituents that are always underrepresented in drafting these standards. In addition, these standards are again voluntary; therefore, there is a real challenge to guarantee that other companies will not introduce rogue digital products and services.

As tech firms continue to push for self-regulation, critics and experts point out that given the high-stakes ethics issues accompanying AI, the industry should not be trusted to self-regulate (Benkler, 2019). To critics, “*self-regulation is not going to work for emerging technologies, because companies that follow ethical guidelines would be disadvantaged with respect to the companies that do not. It's like driving. Whether it is on the left or the right side, everybody needs to drive in the same way; otherwise, we are in trouble*.”

In general, the tech firms have an unmatched influence and power relative to other platforms (public or civil society) in the technology governance debate, except in a handful of jurisdictions, such as EU, majority of jurisdictions especially in emerging economies can barely master a coherent governance strategy when it comes to these firms and their services as highlighted above. Further example include is blockchain-powered platforms such as Binance which operate in multiple countries and is used in trading of billions of dollars in crypto assets without an office or regulatory oversight^5^. Other examples are case of AI-powered platforms such as Airbnb or Uber that are “illegal” in number countries (e.g., South Korea is one such example) but continue to operate in such countries as if those countries legal systems don't matter. Such cases are numerous and it is beyond the scope of this article to discuss these in details.

### User-Centric Organizations Driven Digital Governance

**Figure 3:**
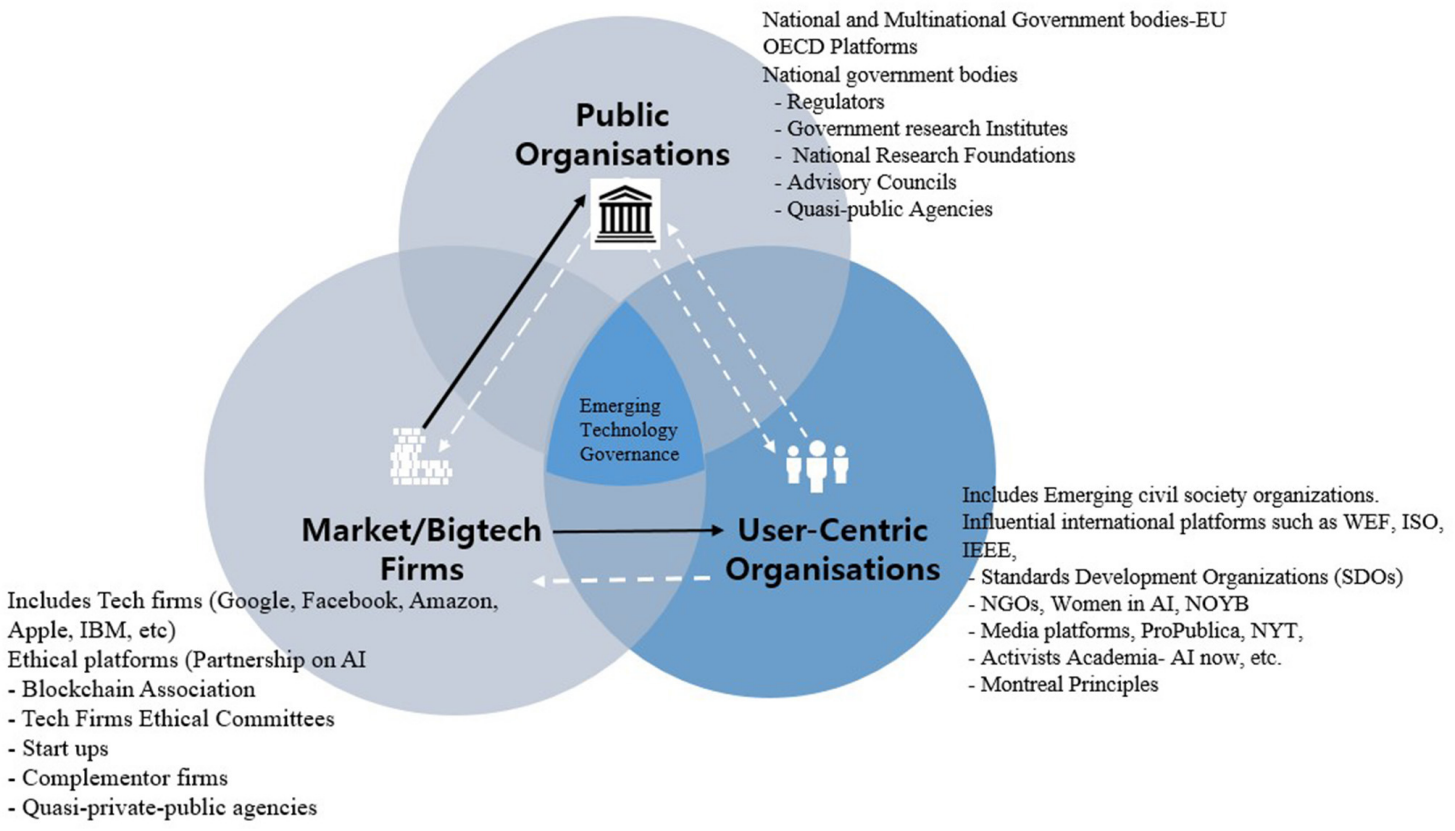
Emerging governance centered in user-centric organizations.

Figure 3 captures the emerging efforts and initiatives of civil society actors to establish policies, standards, and institutional mechanisms that govern digital technologies. The institutional players emerging in this space include the influential International Standards Organization (ISO), the World Economic Forum (WEF), the Institute of Electrical and Electronics Engineers (IEEE), international media houses, and non-government organizations (NGOs), among others. These organizations are playing roles as advocates, quasi-regulators, watchdogs, and policy and standards setters, all of which form building blocks for the global governance of emerging technologies.

As advocates and quasi-regulators, civil society organizations have published over 100 AI principles, such as the Top 10 principles for ethical AI by the UNI Global Union, the Toronto Declaration by Amnesty International and Access Now, and Universal Guidelines for AI by the Public Voice Coalition. These initiatives add weight to the growing importance of mitigating the risks and dangers posed by unregulated and unaccountable adoption of emerging technologies, and point toward regulatory reform and industrial policy in governing new technologies. These efforts have not only brought global attention to the ethical, human rights, and social problems that surfaced due to the increasing application of new digital technology, but also provided critical input for digital policy-making in the public and private sectors. Such efforts have also increased the pressure on industry players to adopt ethics by designing models in their technology development chains.

As a policy- and standard-setter, civil society is emerging as a quasi-regulator; for example, the ISO has drafted standards, such as ISO/IEC JTC 1/SC 42 (ISO, 2017), which focus on responsible adoption and use of AI. Similarly, IEEE, the global association of engineers, has established a global initiative for ethical considerations in AI and autonomous systems (IEEE SA, 2017), publishing standards such as IEEE P7OO4^TM^ Standard for Child and Student Data Governance, IEEE P7005^TM^ Standard for Transparent Employer Data Governance, and IEEE P7006^TM^ Standard for Personal Data Artificial Intelligence (AI) Agent. Although these standards are voluntary, these efforts translate into the civil society by imposing some form of regulatory control on the AI industry, despite it being a weak control due to the scale of standards required for the pervasive adoption of AI.

As watchdogs, civil society players and international media organizations have highlighted the unethical and biased application of emerging technologies developed by the industry, in what has been termed “whipping, naming, and shaming” private and public sectors to rethink about the development and adoption of AI and blockchain products and services. For instance, platforms such as ProPublica, *The New York Times*, and CNN have given global consumers a daily dose of news on the dangers of unethical digital products from tech firms (Murgia and Shrikanth, 2019). Similarly, NGOs such as Women in AI and NOYB (None of Your Business) have called out tech firms and national governments to ignore ethically questionable and human rights—abusing products and services. Such activism and campaigns using mass media platforms have not only increased global consumer awareness but also banned some companies from participating in some markets (Knight, 2019). In some cases, activism and campaigns have become critical tools and levers for “civil society to control the emerging technology industry^6^.” For example, campaigns highlighting issues of AI algorithm bias, discrimination, and racism have resulted in firms such as Google and Facebook issuing apologies (Grush, 2015) and fixing flaws in their algorithms (Gillum and Tobin, 2019).

Activism and complaints focusing on privacy and data protection have influenced governments to intervene through regulation and monetary actions, which have affected tech firms; for example, NOYB complaints resulted in the invalidation of the Safe Harbor agreement—an international agreement designed to transfer European data to the US—and the organization's other campaigns have resulted in monetary fines issued by governments for breach of privacy laws.

Although the above steps are moving in the right direction in terms of effective governance of emerging technologies, an important unanswered question concerns the translation of these standards, principles, and any associated research into practice. How should society be organized to govern disruptive technologies? Who will be responsible for governing these products and services through monitoring, certifying, and approving them? Moreover, how can we ensure that the work of civil society can translate into policy and regulatory action? Relevant activities of civil society have been limited to date. On the one hand, they largely only target ethical and consumer protection in rich countries, resulting in an “ethics and governance divide.^7^” This means that the privacy and data protection in European countries is prioritized by industry due to regulation, while users elsewhere are left to the mercy of domineering BigTech firms (Kelion, 2019); for instance, the “right to be forgotten” applies only to European consumers. Civil society groups have also only pushed industry to respond through ethics washing (Wagner, 2018) as opposed to taking fundamental steps to address the ethical problems posed by their products. Despite many civil society initiatives, these efforts have had a limited impact, barely touching the ethical, legal, and governance challenges associated with the adoption of new digital technologies. Questions are emerging as to whether civil society can ensure the responsible adoption of these technologies. Some proposed models for governance look at the evolution of civil society groups from advocacy and watchdog roles to tech-certification platforms, similar to the case of environmental civil society, as a means of creating sustainable and impactful governance models.

This preliminary review thus suggests that civil society platforms—beyond publishing principles and nudging the tech firms to adopt “ethics by design” have limited enforceable power to translate these principles, guidelines and standards that can be adopted by tech firms. Tech firms in particular have shown disdain and “untouchable” attitude when “ethical and governance issues are raised. A case in point is google rebuffing of these “ethics”—see Google firing its own ethics team for pointing out ethical issues with their AI-powered algorithms (Karen, 2020). Similar allegations have be made against Facebook in the ongoing “Facebook Files” scandal.

### Emerging Digital Governance in the Public Sector

**Figure 4:**
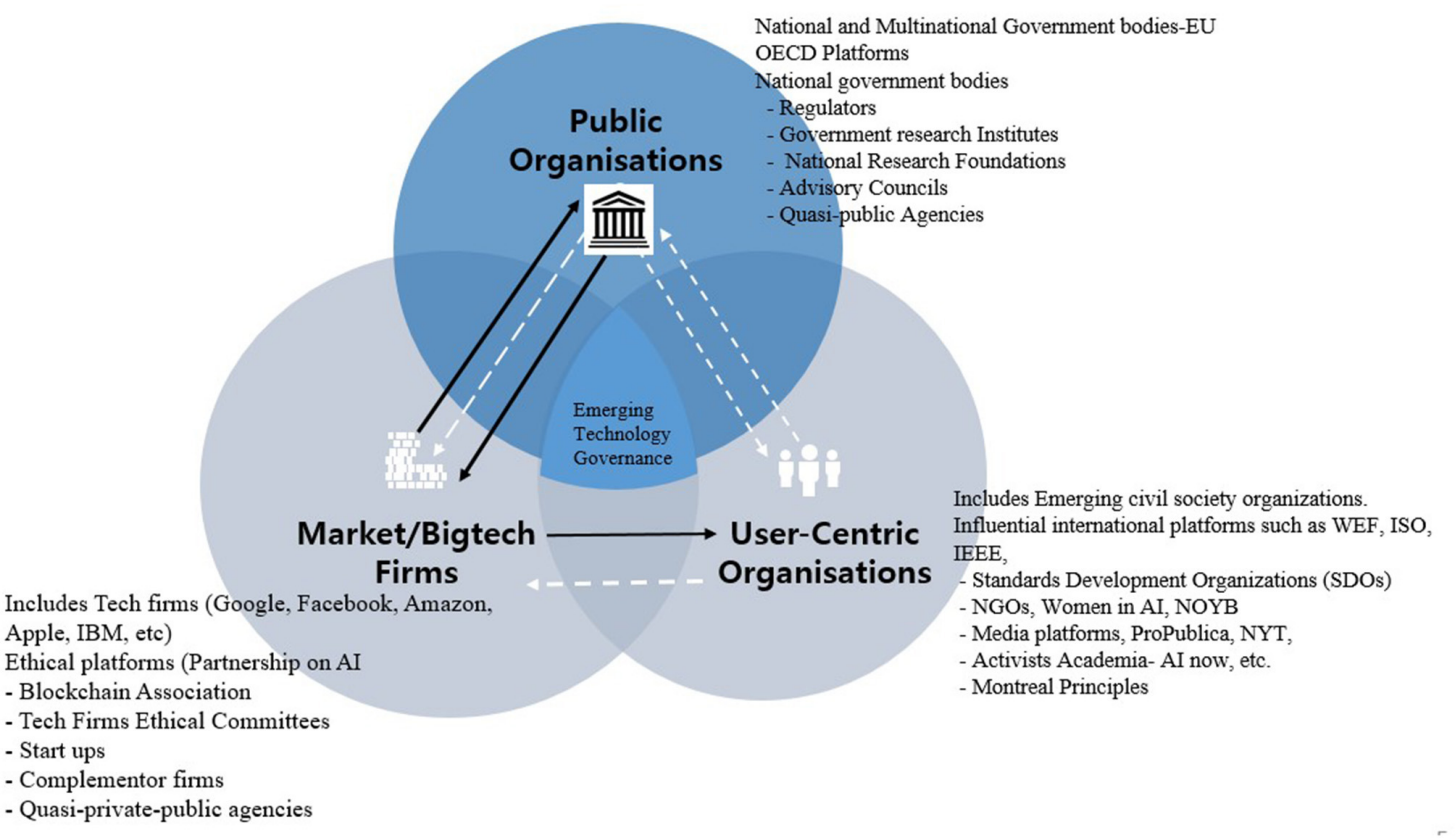
Emerging technology governance platforms in the public sector.

Next, this section discusses emerging tech governance platforms of various governments and tries to untangle their association with other players to promote digital innovations and good governance. These emerging platforms consist of government and inter-government initiatives focusing on policy, institutional, and capacity-building efforts for the governance of emerging technologies (Figure 4). In this model, governments take on complex changing roles as regulators or non-regulators, buyers of digital technologies, innovation policy-makers, taxation bodies, standards- and principle-setters, and self-regulation monitors. A combination of these roles typically helps governments and public agencies achieve the dual objectives of promoting digital innovations and safeguarding the public against the risks and dangers posed by these technologies. Typically, the role(s) of a particular government also dictate the kind of platforms needed to achieve these objectives: emerging governance platforms tend to be divided between innovation-promoting platforms, focusing on innovation and investment in new technologies, and governance platforms, which deal with the risks and dangers of new automated technologies.

**Figure 5:**
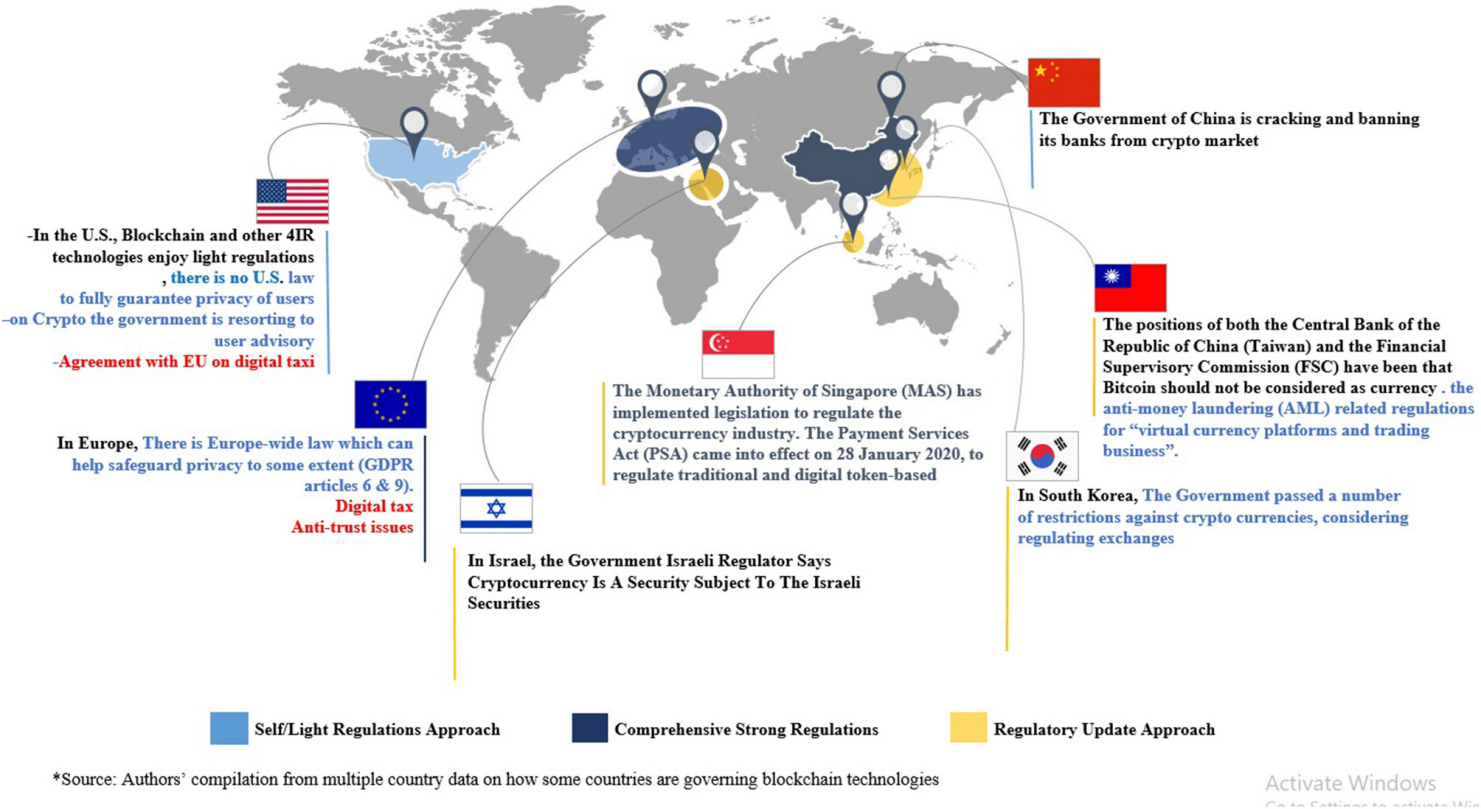
Emerging technology governance ecosystem map of major countries.

In this context, the governance efforts of governments vary from those that exert a strong public hand in promoting innovation to those that leave this role to the market, and from those pushing for comprehensive technological governance regimes with strong laws enforced by national regulatory agencies, who have the authority to investigate and punish regulatory breaches in a given jurisdiction (see Newman, 2012), to others that favor restrained regimes with soft regulation and a limited government role in emerging technology governance. In the latter approach, tech firms are typically left to monitor themselves. Below, we untangle the emerging, complex web of governance platforms within the public sector.

#### Governments as Digital Innovation Policymakers

Many governments view emerging technologies as a new economic growth engine (Kalenzi et al., 2020). The strategies adopted by major countries focus on four main areas: the adoption and diffusion of digital technologies, strategies for collaborative innovation, research and innovation, and digital entrepreneurship (OECD, 2019). Additionally, these vary between countries that advocate strong promotion of digital innovation and those that leave it to the market.

##### National and Multinational Initiatives on AI Governance

Governments of major countries have reached a consensus that the new digital technologies, including AI and blockchain, are a new, endless frontier (Kalenzi et al., 2020). For instance, on AI technologies, governments in developed countries have committed significant investment and developed comprehensive policy agendas to support the development and adoption of these technologies. In China, the government published the New Generation Artificial Intelligence Development Plan, which envisions spending 150 billion dollars to establish China's leadership in AI (Future of Life Institute^8^). China has also put in place institutions to properly execute the policy, such as a new AI promotion office to coordinate their policy and investment plans on AI.

In the US, we see similar initiatives aimed at maintaining America's innovation and technology leadership on critical technologies, including AI and blockchain. In June, the Senate passed a bipartisan 250 billion dollar tech bill to fund advanced research on emerging technologies, including AI, blockchain, and robotics, to maintain US global leadership (Franck, 2021). Moreover, the US enjoys well-funded private investments in digital innovations. Relatedly, the EU has followed the US and China, investing 806.9 billion euros in the “NextGeneration” transnational EU economy, with part of this investment going to promoting AI, digital innovations, and renewable energy (European Commission^9^).

Other governments have also joined the race to develop new economies powered by digital innovations and AI. This year, the government in South Korea launched the Digital New Deal (Yonhap News, 2020), a massive investment and policy strategy to renew the Korean economy on the basis of AI, data, 5G, and other digital technologies. In Singapore, the government established the Digitalizing Singapore agenda; in Australia, the Digital Transformation Strategy with an AU $1.2 billion investment plan targets similar goals as the others (Pash, 2021). Others, such as Canada, Israel, Japan, Switzerland, and France, have similar policies, investment plans, and institutional frameworks to promote the development and adoption of AI and digital technologies.

##### National and Multinational Initiatives on Blockchain Governance

Similar to the AI promotion policies above, governments of major countries are taking a strong interest in developing and adopting blockchain technologies. However, unlike AI promotion policies, most of the investment agenda in blockchain is driven by the private sector and startups in the majority of the countries. On this front, the European Union is one of the leading contenders. For example, for 2016–2019, the European Commission provided over 180 million euros in grants under the Horizon 2020 program to fund the development of blockchain technologies by startups (European Commission^10^). Further, the commission is streamlining the standardization and usage of data, which is critical to developing blockchain and AI ecosystems. Some prominent initiatives include the 4.9 million euro DECODE project^11^ and the 3.4 million euro MyHealthMyData projects (European Commission^12^). Regarding institutional arrangements, Europe established the European Blockchain Partnership (EBP) to develop coordinated efforts to build transnational blockchain infrastructure for public services.

In China, the government is cautiously promoting the development of blockchain/distributed ledger technologies (DLT). In recent years, the Chinese government has launched a National Blockchain-Based Service Network as its leading platform for public and private sector companies to collaborate in developing blockchain technologies.

In the US and UK, blockchain technology development is driven by private sector investment, BigTech, and startups. They together represent the biggest innovators and promoters of blockchain technologies. On a smaller scale, similar initiatives are going on in Korea, Singapore, and Canada, among others.

The preliminary lesson from the above diverse policy agendas is that when it comes to promoting AI, blockchain, and other digital technologies, the main concern is whether a country can get and maintain a competitive advantage, or whether it will stagnate in the lower ranks of global value chains. Even though the execution of the policy will differ from country to country—from those that favor a strong government hand (China, Korea) to those that favor a mixed public and private approach to those that are mainly leaving the market to take the lead—there is no doubt that major countries are taking solid action to renew their economies on the pillars of AI, blockchain, data, and digital technologies.

### Governments as AI and Blockchain Regulators

As mentioned above, there is broad agreement on a policy agenda to promote the development and adoption of AI, blockchain, and other digital technologies across countries. There is also, however, broad agreement that these technologies increase monopolies, pose privacy risks, increase inequality, endanger democracies, and increase misinformation, fraud, bias, safety issues, and that these issues call for regulatory mechanisms to limit the negative impacts of these technologies.

Despite the policy consensus, there are vast disagreements across governments, the private sector, and civil society organizations on how to achieve optimal regulation to mitigate these negatives. The only exception to these disagreements is the recent global agreement on digital taxes. If we consider cryptocurrencies, for example, many of them offer cross-border payments and trading applications, among other applications. Given the multi-national operations of cryptocurrency platforms, many would expect players, especially in the public and private sectors, to agree on common rules and standards to regulate the industry. In fact however, where some major countries, such as China, have recently imposed bans on all transactions of cryptocurrencies (BBC, 2021a), and others, such as South Korea and Japan, are proposing strong regulatory mechanisms to regulate cryptocurrencies and blockchain technologies, others, such as the US and UK, are taking softer approaches such as consumer advisories and fines. As a result of these disagreements, the effective regulation and hence also development and adoption of blockchain cross-border applications, including in healthcare (COVID-19 digital certificates), supply chains, payments, and so on faces significant challenges.

The disagreements are more pronounced in the governance of AI and related digital technologies. On this front, countries are taking different approaches, with some favoring strong and comprehensive regimes, such as the EU and China, and others taking light/soft strategies (Floridi, 2018), such as the US and UK; still others taking a mixed approach; and the majority belong to the wait-and-see camp. To illustrate, in Europe, besides the well-known GDPR, which regulates privacy and data-sharing (Albrecht, 2016), the European is proposing new rules and policy actions aimed at furthering the responsible development of AI. These include communication to foster a European approach to responsible AI, a coordinated plan for member states, and the Artificial Intelligent Act, which lays down harmonized rules to regulate AI. Still, even within Europe, we see that the UK has so far taken a softer approach to AI regulation. For example, the UK Center for Data Ethics and Innovation was established to oversee, but not regulate, AI and data-driven digital technologies. But recently, there have been ongoing discussions on a proposed Online Safety Bill, which will regulate data and AI-driven applications when passed.

On one extreme in this debate is the US, which primarily embraces market-driven “self-regulation” and has no comprehensive data and privacy regulations, except California's AB 5 Law (Bukaty, 2019) and San Francisco's banning of AI-powered facial recognition technologies, somewhat ironical given its status as the center of the US tech industry, including blockchain and AI. Beyond these, the US largely relies on light approaches: principles, standards, self-regulation, and generally non-binding guidelines. The absence of regulations has forced private companies and user-centric organizations such as media groups to “step into the breach” and defend consumers from actual and potential harmful effects of AI. For example, Apple has recently upgraded privacy settings on all its devices to give users more power over their data. Relatedly, media organizations such as *The New York Times, The Wall Street Journal*, ProPublica, among others, are pursuing a sustained campaign to alert users in the US and globally of abuses and dangers of AI-powered applications. However, the effects of these efforts and whether they can lead to fundamental changes to mitigate the risk of AI remains in question.

The broader take away from public efforts in the governance debate is that much as many governments have a better grasp of innovation policies to promote the technologies. However, this article agrees with (Cusumano et al., 2021) that many governments (except in handful of jurisdictions such as Europe and China) are relatively weak to govern emerging technologies. “*Most do not have the skills, or resources, to regulate and monitor the dynamic, on-going changes with digital platforms and their complex technologies and operations.”*

### Multi-National Platforms for Governing AI and Digital Technologies

Because of these weakness, most countries (especially developing countries), have taken a wait-and-see approach and remained silent on the question of governing emerging technologies. Recently, however, this debate is taking on a more multi-national dimension. Some countries are grouping to try and shape regulation and responsible development of AI and data-driven technologies. For instance, in May 2019, OECD member countries and non-member countries such as Argentina, Brazil, and Romania signed and adopted AI principles (Budish and Gasser, 2019) which, although non-binding, sends a strong message to the private sector that these countries want responsible development and adoption of AI technologies. OECD member countries and the US and Poland have followed up by establishing the Global Partnership on Artificial Intelligence (GPAI); this newly established platform aims to advocate for principles for responsible stewardship and global coordination of national policies and international cooperation for trustworthy AI. It remains to be seen whether such efforts will lead to a global agreement on standard and enforceable rules on responsible development and adoption of AI; it may remain difficult, given competing interests, disagreements on how to regulate AI, the technology race to develop AI, and technology-hegemonic struggles among major “developer” nations.

Worse still, these multi-national groupings are essentially “rich nations' clubs,” exclusive groups that have left the Global South on the sidelines of the AI governance debate.

## Is There Common Ground?

In this overview, we consider disparate emerging platforms from the perspectives of public, private, and civil society. Based on our preliminary analysis, we can clearly see that there is a convergence of interests among all parties in promoting the responsible development and adoption of emerging technologies. For example, as shown, all three major players have established technology principles and codes, have developed or are in the process of developing standards, and have established a framework to realize these. In industry, we see the development of ethics committees; in government, we see rise in advisory committees and digital innovation watchtowers; and in civil society, we see the rise of advocacy groups.

In principle, these three groups of actors—firms, government, and civil society—want similar things. However, their diverse interests appear in their underlying motivations, and emerge as they take action to shape the rules governing emerging technologies. In this study, we argue that this might be the key to understand the shaping of technology governance and its possible future. Ultimately, the question of responsible development and adoption of emerging innovations is a question of the public (represented by the public sector and civil society) vs. the market, and a question of whether industry will put the interests of users ahead of profit. It is a question that turns on whether governments have the resolve to overcome pressures from tech firms and the digital innovation race to safeguard the interests of the public. Finally, there is the question of whether civil society in the emerging technology governance space can generate the clout to translate advocacy into action.

In this preliminary analysis, we show that the current motivation for industry is profit and that critics might be correct in suggesting that industry actions, such as establishing powerless ethics committees and technology principles, are classic examples of ethics washing or paying lip service while, in fact, doing everything possible to profit from these new, flawed yet powerful technologies (e.g., facial recognition technologies, ICOs, fraudulent cryptocurrencies, participation in military AI projects).

### Emerging Self-Regulation Platforms

Our overview shows that there is a convergence of interest among nations to promote emerging innovations. For example, all major countries have published digital innovation policies and followed them up with concrete investment plans to develop new technology capabilities. Similar principles have been adopted by the OECD and the G20, including the US. However, governments diverge in terms of how they develop and apply safeguards and laws to protect users from the unethical use of these powerful innovations. Countries with tech firms that have vested interests in digital innovation, such as China and the US, are opting for self-regulation and softer regimes. In the case of the US, hard standards, such as the California consumer privacy law and San Francisco's banning of AI-powered facial recognition technologies, do exist at the state level (and have generated global buzz). In the foreseeable future, self-regulation platforms are likely to consolidate in countries with large tech sectors.

### Comprehensive and Strong Government Platforms

Among the rest of the world, Europe is taking the lead to promote innovations (policies and funding), while simultaneously regulating tech firms through laws and codes of conducts relatively strictly. It can do this as it has recognized market power, and there are serious incentives to promote and protect the budding tech industry in some European countries. However, it is unlikely that most developing countries and other countries in “wait and see group” can exert any serious regulatory or governance effort—certainly not on the scale of GDPR, which may upset tech firms. A case in point is the recent ruling on the “right to be forgotten” in the case against Google, which stipulated that the GDPR applies in Europe but not in other countries. Europe, therefore, is likely to remain a bedrock of comprehensive regulations, while most other countries, especially developing ones, remain as laggards (running wait-and-see platforms) in the emerging technology governance space.

### Soft Law Platforms

Between these two sides, self-regulation and strong government regulation, lie many middle powers, such as Singapore, France, Sweden, and New Zealand, with actions such as grand digital innovation funding schemes, governance frameworks, advisory committees, Canada–France–New Zealand, and a mixture of strong regulations such as France's digital taxation policy and Singapore's anti-fake news laws with market–friendly soft regulations in several places. We suggest that the motivation for these varies. Singapore, which is Asia's hub for AI BigTech firms and is quite economically dependent on their presence, is between a rock (rubbing BigTech the wrong way) and a hard place (protecting users); hence, its middle ground actions should be interpreted in that light. France, Sweden, and much of Europe may feel left behind in the digital technology race and, thus, may be eager to adopt aggressive policies to promote innovation while taking protective measures against tech firms.

We also show that civil society groups largely agree with industry and public sector actors on principles and standards. However, beyond publishing principles, advocacy, and calling out BigTech firms, civil society is in an unenviably weak position. Its actions are at best reactive, and although its organizations have proposed principles and values, they have no teeth. This is akin to having laws or values without the means to implement them. Thus, we propose that for civil society to be effective, it will have to evolve and develop stronger certified platforms.

## Conclusion

In this study, we survey the approaches of different players to the governance of emerging digital technologies, with a focus on AI and blockchain. Our preliminary analysis suggests that the different relevant parties are just beginning to come to terms with the governance challenges that emerging technologies pose for society. Within industry, we see the emergence of entities pushing for self-regulation, such as the PAI and the Blockchain Association; formation of ethics committees; published principles; and emerging global collaboration on standards and ethical frameworks. Conversely, within civil society, we see a rise in advocacy and the need for standard platforms that call for responsible development and adoption of emerging technologies, where different governments would establish platforms for promoting innovations and responsible adoption of new digital technologies.

There appears to be a broad consensus on the need for innovation policies and the establishment of effective principles, standards, and frameworks for responsible digital innovation. However, here, we have shown that firms, governments, and civil society are driven by different motivations and vested interests; we believe these differences will be a challenge to a broad consensus on the governance of these new technologies.

Due to the different motivations, governance platforms will evolve and mature or stagnate depending on the environment. In some environments, such as in the US, where BigTech firms have a dominant presence, governance issues will likely take a back seat, and we may see a consolidation of self-regulation approaches, which are apt to focus on profits and their stakeholder's interests (“Regulations stifle innovation narrative”). In consumer protection–oriented environments, such as Europe, there will likely be a consolidation of comprehensive approaches to regulate and promote digital innovations that protect consumers as well as budding industry, to grow Europe's own digital technology base. Amid such a power play, civil society players will likely remain insignificant in the emerging technology governance space unless there is a shift to a more active and stronger civil society.

It is now widely accepted that these emerging technologies represent present and future economic growth engines, but also pose significant risks to society. Given the lack of global consensus on how to mitigate emerging problems, we suggest that it is up to each country to erect safeguards in each domain. This is particularly true in healthcare, security, public safety, and transportation, where digital technologies present significant potential but also serious bias, safety, and privacy risks. Immediate safeguarding mechanisms should be implemented to protect consumers from the rogue and unaccountable use of digital technologies. One good example of such safeguards is the proposed FDA Medical Device action plan, which puts in place measures such as the total product life cycle (TPLC) approach for AI and ML medical device safety.

Other safeguards could be implemented following the example of Britain's Center for Ethics and Innovation, which has as its mission to identify how users can enjoy the full potential benefits of data-driven technology within the ethical and social constraints of liberal democracy. By taking on this mission, players, especially governments, could avoid the command and control narrative favored by some (in government, civil society, and even industry) or the self-regulation favored by others, especially in the tech industry. Instead, players could focus on the practicalities of how emerging technologies can be feasibly governed, how to build trust across cooperating entities, how to figure out where to start to deal with ethical and social issues, and how to work together for optimal governance and regulation.

Finally, governments and the private sector could use their buying and licensing powers especially for large AI and blockchain projects such as city-wide facial recognition projects and shipping supply chains, to push industry to adopt ethics by design principles. Beyond this, they could put in place sufficient guidelines and principles for responsible agencies, such as police and security agencies, on the safe and ethical use of these technologies.

For further research, We use the representative cases of AI and Blockchain technologies in the emerging technology governance even though they are different technologies, with different origins and maturity levels because these technologies are some of the leading forces shaping every sector of public and private lives in the digital era. For instance, they are now used more universally, e.g., these days AI is in “virtually everything” but most associated with AI-powered recommender systems that power every leading platform such as Facebook, Google, Amazon, etc. Similarly, blockchain technologies are known powering the current crypto-currency mania but is also applied in numerous other use cases across the globe. Everyday users across the world, enjoy the benefits and conveniences of these technologies but also suffer collectively when these technologies are abused as earlier mentioned. This fact withstanding, further research could focus on one of the two technologies more thoroughly. For instance, the ongoing debate around governing blockchain technologies and applications such as crypto-currencies' issues such as fraud and privacy can benefit from similar earlier studies in the AI space.

The other question that merits further research: How can the different governance platforms in the public, private sector and civil society collaborate to create better governance systems, considering their relative positions?

## Author Contributions

All authors contributed equally to the conception and design of the study, as well as researching, drafting, revisions, and approving the submitted version.

## Conflict of Interest

The author declares that the research was conducted in the absence of any commercial or financial relationships that could be construed as a potential conflict of interest.

## Publisher's Note

All claims expressed in this article are solely those of the authors and do not necessarily represent those of their affiliated organizations, or those of the publisher, the editors and the reviewers. Any product that may be evaluated in this article, or claim that may be made by its manufacturer, is not guaranteed or endorsed by the publisher.

## Footnotes

^1^“Governance refers to processes of governing; or changed conditions of ordered rule; or new methods by which society is governed” (Rhodes, 2012). In this article, we expand on this definition in context of emerging technology governance to include all initiatives aimed at technology promotion and mitigating risks that these technologies pose to society.

^2^“Nevertheless, even A.I. researchers like me recognize that there are valid concerns about its impact on weapons, jobs, and privacy. It's natural to ask whether we should develop A.I. at all” (Etzioni, 2017).

^3^According to Google, “AI has now become a real-world application technology and part of every fabric of society. Harnessed appropriately, we believe AI can deliver great benefits for economies and societies” (Google, 2019).

^4^According to the World Bank: “Civil society... refers to a wide array of organizations: community groups, non-governmental organizations [NGOs], labor unions, indigenous groups, charitable organizations, faith-based organizations, professional associations, and foundations”; for definition see, https://www.weforum.org/agenda/2018/04/what-is-civil-society/.

^5^The Wall Street Journal, $76 Billion a Day: How Binance Became the World's Biggest Crypto Exchange: The trading platform surged by operating fromnowhere in particular–without offices, licenses or headquarters. Now governments are insisting on taking some control. WSJ News. November 11, 2021. https://www.wsj.com/articles/binance-became-the-biggest-cryptocurrency-exchange-withoutlicenses-or-headquarters-thats-coming-to-an-end-11636640029 (accessed November 23, 2021).

^6^“In some instances, civil society campaigns are critical social controls of industry.” For detailed discussion see Christopher et al. (2012).

^7^We define the “ethics and governance divide” as a situation where technology ethics and governance considerations are determined based on location of implementation. Richer and stronger countries for example have strong ethics and governance systems in place in comparison to less developed countries, hence the divide.

^8^Future of Life Institute. *AI Policy China*. https://futureoflife.org/ai-policy-china/.

^9^European Commission. *Recovery Plan for Europe*. https://ec.europa.eu/info/strategy/recovery-plan-europe_en#nextgenerationeu (accessed October 28, 2021).

^10^European Commission. *Blockchain Funding and Investment*. https://digital-strategy.ec.europa.eu/en/policies/blockchain-funding.

^11^See Decode project. https://decodeproject.eu/.

^12^European Commission. *My Health - My Data*. https://cordis.europa.eu/project/id/732907 (accessed June 30, 2021).
